# Genotoxic Damage to Glioblastoma Cells Treated with 6 MV X-Radiation in The Presence or Absence of Methoxy Estradiol, IUDR or Topotecan

**DOI:** 10.22074/cellj.2016.3738

**Published:** 2015-07-11

**Authors:** Nazila Eyvazzadeh, Ali Neshasteh-Riz, Seyed Rabee Mahdavi, Afshin Mohsenifar

**Affiliations:** 1 Radiation Research Center, Faculty of Paramedicine, AJA University of Medical Sciences, Tehran, Iran; 2 Department of Radiology, Faculty of Allied Medicine, Iran University of Medical Sciences, Tehran, Iran; 3 Department of Medical Physics, Faculty of Medicine, Iran University of Medical Sciences, Tehran, Iran; 4 Research and Department Nanozino, Tehran, Iran

**Keywords:** DNA Damage, HIF-1Alpha, 2-Methoxyestradiol, Topotecan, Picogreen

## Abstract

**Objective:**

To explore the cumulative genotoxic damage to glioblastoma (GBM) cells, grown as multicellular spheroids, following exposure to 6 MV X-rays (2 Gy, 22 Gy) with or without, 2- methoxy estradiol (2ME2), iododeoxyuridine (IUDR) or topotecan (TPT), using the Picogreen assay.

**Materials and Methods:**

The U87MG cells cultured as spheroids were treated with 6 MV X-ray using linear accelerator. Specimens were divided into five groups and irradiated using X-ray giving the dose of 2 Gy after sequentially incubated with one of the following three drug combinations: TPT, 2-ME2/TPT, IUDR/TPT or 2ME2/IUDR/ TPT. One specimen was used as the irradiated only sample (R). The last group was also irradiated with total dose of 22 Gy (each time 2 Gy) of 6 MV X-ray in 11 fractions and treated for three times. DNA damage was evaluated using the Picogreen method in the experimental study.

**Results:**

R/TPT treated group had more DNA damage [double strand break (DSB)/single strand break (SSB)] compared with the untreated group (P<0.05). Moreover the R/ TPT group treated with 2ME2 followed by IUDR had maximum DNA damage in spheroid GBM indicating an augmented genotoxicity in the cells. The DNA damage was induced after seven fractionated irradiation and two sequential treatments with 2ME2/IUDR/TPT. To ensure accuracy of the slope of dose response curve the fractionated radiation was calculated as 7.36 Gy with respect to α/β ratio based on biologically effective dose (BED) formulae.

**Conclusion:**

Cells treated with 2ME2/IUDR showed more sensitivity to radiation and accumulative DNA damage. DNA damage was significantly increased when GBM cells treated with TPT ceased at S phase due to the inhibition of topoisomerase enzyme and phosphorylation of Chk1 enzyme. These results suggest that R/TPT- treated cells increase sensitivity to 2ME2 and IUDR especially when they are used together. Therefore, due to an increase in the level of DNA damage (SSB vs. DSB) and impairment of DNA repair machinery, more cell death will occur. This in turn may improve the treatment of GBM.

## Introduction

One of the most prevalent and deadly tumors of the brain is glioblastoma (GBM) multiforme. Its prevalence is mostly found in people of 45 to 65 years of age. It is considered to be among the primary tumors of the central nervous system and is more common among Americans and Africans with male to female ratio of 3:2 and incidence rate of ≈ 5/100000 (1, 2).

Lack of an effective treatment has diminished hope for the survival of patients from this deadly tumor. To treat the tumor various methods such as surgery, radiotherapy, chemotherapy and radio-chemotherapy have been used but the results have not been satisfactory yet (3). For instance, chemotherapy is often toxic to bone marrow and radiotherapy, delivered by external beam, has also been shown to be toxic to normal brain tissues and the marrow. In radiotherapy, the dose which can be administered is severely limited by its ill-effects in normal tissue. Thus use of an appropriate dose to preserve the viability of bone marrow tissue is very crucial, hence radiotherapy alone has not been very effective (2). Chemotherapy is employed to destroy cancerous cells, however, to the level that toxicity of drug would be tolerated by normal tissues (4). Therefore, new methods should be explored to produce safe and effective results. The use of radiosensitizers like 2-methoxy estradiol/iododeoxyuridine/topotecan (2ME2/IUDR/TPT) combined with fractional radiotherapy is among the important techniques for achieving this goal all of which were employed concurrently in this study.

TPT derived from the Chinese tree Camptotheca acuminata with the commercial name Hycamtin is a radiosensitizer agent and used recently as a chemotherapeutic drug (5, 6). This compound is used in a vast array of cancers such as ovarian cancer, lung cancer, leukemia, non-Hodgkin’s lymphoma, myelodysplastic syndrome, melanoma, colorectal and has been recently tested on GBM on both children and adults (6, 7).

TPT stops the cell cycle through inhibition of topoisomerase I (Topo-I) enzyme activity. Inhibition of this enzyme will lead to phosphorylation of DNA which in turn increases both DNA-Topo I complexes and double strand breaks (DSB), and finally leads to apoptotic death (3, 6, 8).

The reduction of Topo-I, due to the activity of P53 protein in wild-type (U87) cells, is seen 2 hours after the start of TPT treatment (6). An *in vivo* study showed that in radio-chemotherapy best results could be achieved when TPT is injected 2-4 hours prior to radiation. On the other hand, injection 2 hours after the radiation did not produce a noticeable effect (8). The Canadian National Cancer Society has evaluated TPT toxicity in a large group of patients and determined the tolerable dose of 2.3 mg/m2 (5 μM) for less than 3 days and 0.5 mg/m2 (1.1 μM) for a one-month-long treatment (5 days a week) (9). Tomicic et al. (6) also applied identical TPT concentration of 1-4 μg/ml for short exposure periods (up to 24 hours) in GBM cells. In U87 glioma cells, 1 μM concentration of TPT can cause phosphorylation of P53, which leads to sensitivity of TPT (10). Combination of TPT with [^131^I] meta-iodobenzylguanidine ([^131^I] MIBG) has been useful in treating neuroblastoma *in vitro* and *in vivo* and addition of TPT simultaneously or after addition of [^131^I] MIBG can increase the cell uptake of TPT (3).

TPT liposome with tamoxifen and wheat germ agglutinin will increase the transfer rate of the drug from the blood-brain barrier and penetration in tumor, and result in reduction of tumor volume and hence more benefit in the treatment. This experiment has been carried out as *in vitro*-*in vivo* in glioma cells of the brain (11, 12). In order to increase the survival period of GBM patients, research has shown that combining radiotherapy with TPT could result in an increase of 1-2 years in survival rate which is a 16% increase (7).

IUDR was used as a radiosensitizer early 1960s and has replaced thymidine for DNA replication and in reaction with hydrated electrons infused by ionized rays, produces reacting uracil free radicals and halide ions, and finally causes single strand break and subsequently DSB in DNA thus causing cellular death (13, 14).

Hypoxia inducible factors (HIF-1a) is a heterodimer transcription factor responsible for stopping cell cycles in endothelial cells at G0/G1 phases; its activity is inhibited by methoxy estradiol and this in turn leads to inhibition of cell cycle checkpoints. Experiments have shown that damages caused by ionizing irradiation following sensitization by IUDR in spheroids cells treated with 2ME2 is far more effective than employing IUDR alone (13, 15).

Six MV X-ray was used in this research. One of the reasons for using this kind of ray was the ability to control the output. With the collimation system, excess radiation can be controlled for healthy tissues surrounding the tumor and dose optimization can be improved (16).

In this study TPT was used along with IUDR and 2ME2 as radiosensitizer agents to explore their combination for a possible cumulative effect and improvement in tumor treatment.

Three-dimensional culture systems are closer to tumor behavior and consist of three layers of anoxia, hypoxia and normoxia. The use of these multicellular spheroid models will also reduce the need for whole-animal studies in order to mimick small tumors and micrometastases (17).

We used the Picogreen method as a suitable tool to determine the single strand break (SSB)/DSB ratio and as a sensitive probe for determing the level of damage to DSB. Its advantages include the following: i. it was an easy and fast method, ii. required only a few samples , iii. a sensitive method with high accuracy, iv. it had a vast range of manifestation, v. it did not become imbalanced because of cell metabolism due to nano-matter , vi. strong signal to nuance and vii. it can be applied to frozen samples (18, 19). Cells cultures were stained through Picogreen which acted as a fluorescent color. The Picogreen binds to DNA threads with DSB breaks and when we denatured DNA by alkaline buffer, the free Picogreen in a given time was equivalent to SSB and DSB in DNA. To quantify the results obtained from DNA damages, we used plasmid emancipated from bacteria, in the manner that all DNA threads were destroyed by the DNase enzyme (20, 21). We then added Picogreen and measured fluorescence intensity. Despite the research done on the effect of TPT on cancer cells of different organs, little information is available regarding the mechanism of changes in cell nuclei. On the other hand, research has shown that DNA as a target molecule plays an important role in the process of cellular damage.

## Materials and Methods

### Cell line

In the experimental study, human GBM cell line U87MG was purchased from the Pasteur Institute of Iran and cultured in minimum essential medium (MEM, Gibco/Invitrogen, USA) supplemented with 10% fetal bovine serum (FBS, PAA/Austria), 100 U/ ml of penicillin streptomycin (PAA/ Austria) and 20 U/ml of fungizone (Gibco/Invitrogen, USA).

### Spheroid culture

Spheroids were cultured using the liquid overlay technique. A total 5×10^5^ cells were seeded in 100 mm T-25 flasks (NEST/Austria) coated with a thin layer of 1% agar (Sigma/Aldrich, Germany) with 10 ml of MEM supplemented with 10% FBS. The plates were incubated at 37˚C in a humidified atmosphere and 5% CO_2_ (memmert, Germany). Half of the culture medium was replaced with fresh culture medium every three days to feed the cells.

### Growth curve

To draw spheroid growth curve, one spheroid cell was seeded per well in multi-well plates coated with a thin layer of 1% agar and 1 ml of MEM. The multi-well plate was incubated at 37˚C in a humidified atmosphere and 5% CO_2_. For 28 days, at 72 hours intervals, the vertical diameters of cells from triplicate were measured by a microscope. Next, cell volumes were calculated according to the formula V=a.b^2^.π/6 where a is the small diameter of the cells, b is the large diameter of the cells and V is the volume of spheroid cells. An average of nine counts was used to define each point [mean ± standard error mean (SEM)]. Half of the culture medium was replaced with fresh medium twice per week. Then the growth curve was plotted. In the linear area or logarithmic phase of the curve, the cell volume follows the formula V=V_0_×e^kt^ where V_0_ is the initial volume of the cells, V is the volume of the cells after time t and k is the gradient of the logarithmic phase of the curve. The volume doubling time (VDT) of the cells was then determined according to the gradient of the logarithmic phase of the curve.

### Drug treatment and cell irradiation by 6 MV X-ray linac

Glioma cancer cells were grown on a layer as 3 dimensional spheroid cells in a liquid media with 300 μm diameters. Then irradiated cells with Xray were treated with three types of drugs including: 2ME2, IUDR and TPT.

In this study, 6 groups of growth cultures were set-up:

1 Control group
2 Group not treated (this sample was irradiated)
3 Group treated with TPT
4 Group treated with 2ME2/TPT
5 Group treated with IUDR/TPT
6 Group treated with 2ME2/IUDR/TPT

Groups 2-5 were irradiated once with 2 Gy dose of 6 MV X-ray. Group 6 was treated sequentially with 2- 2ME2 for 1 VDT, IUDR for 1 VDT TPT for 2 hours, then one flask was irradiated once with a 2 Gy dose of 6 MV X-ray and eleven flasks were treated for three times during this period and irradiated with total of 22 Gy dose (each time 2 Gy) of 6 MV X-ray in 11 fractions. The concentration of drugs were 250 μM, 1 μM and 1 μg/ml, respectively in MEM containing 10% FBS (22, 10). At the end of the exposure, the DNA damage was evaluated using the Picogreen method.

### Picogreen assay

Radiation-induced SSB and DSB in the DNA of GBM cells were evaluated using the Picogreen assay according to the protocol by Schroder et al. (23).The solution used to denature DNA was prepared as follows:

fluorescent dye stock solution was the Picogreen dsDNA quantitation reagent (solution A, Life Technology/Invitrogen, USA). Calcium and magnesium-free phosphate buffered saline (PBS, Ca/Mg-free PBS) consisted of 137 mM Nacl, 2.7 mM KCL, 4.3 mM Na_2_HPO_4_ and 1.5 mM KH_2_PO_4_ (solution B, Gibco/Invitrogen, USA). The lysing solution contained 9.0 M urea, 0.1 % sodium dodecyl sulfate (SDS) and 0.2 M ethylene diaminetetraaceticacid (EDTA) at pH=10 with NaOH (solution C, Sigma/Aldrich, Germany). Lysing solution supplemented with Picogreen consisted of 10 μL of the original stock dye/ml of solution C (solution D, Life Technology/Invitrogen, USA). The EDTA solution contained 20 mM EDTA (solution E, Sigma/Aldrich, Germany). NaOH stock solution consisted of 1.0 M NaOH and 20 mM EDTA (solution F, Gibco/Invitrogen, USA). Working NaOH solution was prepared fresh prior to use. A total of 2 mL of solution F was added to 18 mL of solution E with pH at 12.40 ± 0.02 to create solution G.

In order to determine the DSB induced in GBM cells, 3 tubes with 50,000 cells/mL for each group were prepared with 300 μl of solution C and 300 μl of solution D. To lyse the cells, all cell groups were placed in the dark for 40 minutes. The amount of DSB were determined by measuring fluorescence intensity for each cell group with a spectroflourometer (Shimadzu/USA) at 485 nm excitation and 528 nm emission wavelengths.

Next, 50 μl of solution G was added to 600 μl of lysed cells in each group (control, irradiated and treated+irradiated). The amount of SSB was determined after three hours by measuring fluorescence intensity in each group.

### Preparing the calibration curve

To determine the amount of fluorescence intensity for digestion of all DNA in cells, various concentrations of DNase combined with 300 μl of solution D and variable volume of PBS (resulting in the final volume of 800 μL) were added to various concentrations of non-irradiated intact GBM and lysed cells (50,000 cells/ml solution C).

### Statistical analysis

Statistical analysis was performed using independent-samples t test and ANOVA followed by scheffe test as the post-hoc analysis by SPSS software version 16. P<0.05 was considered to be significant.

## Results

### Spheroid culture

**Fig.1:**
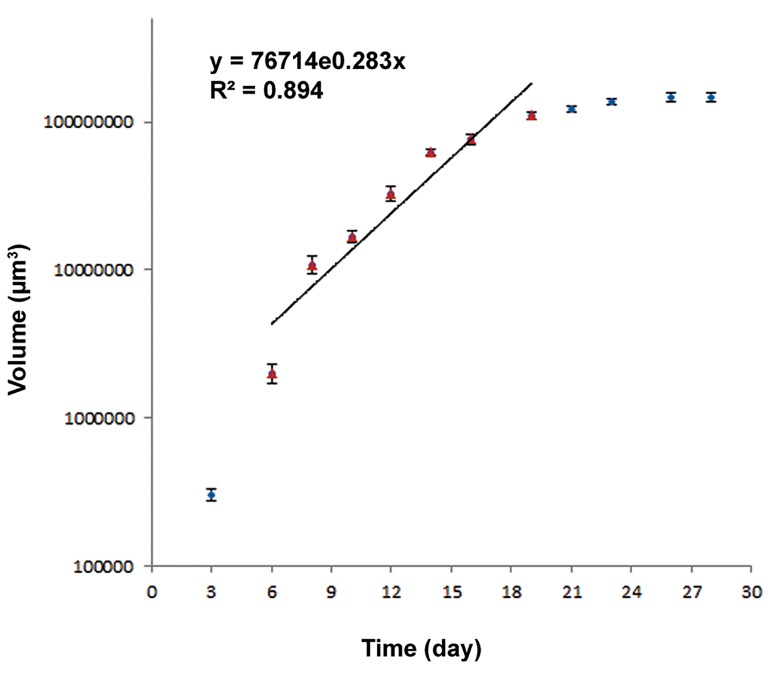
Growth curve of U87MG cell line in spheroid cultures. An average of nine counts was used to define each point. Mean ± SEM of three experiments is shown on the curve. y; Volume (μm3), x; Time (day), R2; Regression coefficient and SEM; Standard error mean.

U87MG cells were turned into spheroids in liquid overlay cultures. The VDT of these spheroids was approximately 58.77 hours which was applied as the drug treatment time for cells (Fig.1).

### Double strand break and single strand break in DNA molecules

The Picogreen assay was used to determine the prescence of induced SSB and DSB in DNA. The spectrum obtained at 300 μm from samples of the irradiated ,treated and control groups of U87MG cells, and spheroids treated with Picogreen solution were measured at an excitation wavelength of 485 nm and emission wavelength of 530 nm (taken by a Shimadzu spectroflourometer). The average fluorescence intensity in the control and treated groups for GBM cells with 2 Gy and total dose of 22 Gy (each time 2 Gy) of 6 MV Xray in presence of 2ME2, IUDR TPT are shown in tables 1 and 1. Table 1 shows fluorescence reduction in the treated and irradiated groups. Whenever radiation and treatment processes had to be repeated, on one hand we observed an increase in DSB and on the other hand a reduction in SSB in DNA (Table 2).

**Table 1:** Measured average fluorescence intensity of control GBM cells and those irradiated by 2 Gy X-ray 6 MV, to determine the amount of SSBs and DSBs in presence of 2ME2, IUDR and TPT


Group	Mean fluorescence nm (DSB)	Mean fluorescence nm (SSB)

Control	595.72± 14.38	496.64± 10.04
R	514.12± 14.28	455.84± 7.48
R+TPT	485.22± 3.74	442.24± 7.48
R+2ME2+TPT	496.92± 7.48	429.66± 8.10
R+IUDR+TPT	456.32± 7.48	411.64 ± 7.48
R+2ME2+IUDR+TPT	437.52± 10.88	388.52± 6.80


GBM; Glioblastoma, SSB; Single strand break, DSB; Double strand break, 2ME2; 2-Methoxy estradiol, IUDR; Iododeoxyuridine, TPT; Topotecan and R; Radiation.

**Table 2:** The measured average fluorescence intensity of irradiated GBM cells by total does of 22 Gy (2Gy/fraction) with 6 MV X-ray in presence of 2ME2, IUDR and TPT (thrice) , to determine the amount of SSB and DSB


Fraction	1	2	3	4	5	6	7	8-11
Total dose (Gy)	2	4	6	8	10	12	14	16-22

Mean fluorescence(nm, DSB)	437.52± 10.88	416.54± 9.86	389.00 ± 8.50	357.72 ± 14.28	318.96± 10.54	270.34± 12.24	257.00 ± 9.18	257.16± 12.07
Mean fluorescence(nm, SSB)	388.52± 6.80	408.24± 10.71	431.30 ± 9.52	455.84 ± 15.30	480.32± 6.46	493.92± 6.80	496.64 ± 6.80	496.16± 6.18


GBM; Glioblastoma, SSB; Single strand break, DSB; Double strand break, 2ME2; 2-Methoxy estradiol, IUDR; Iododeoxyuridine and TPT; Topotecan.

**Fig.2:**
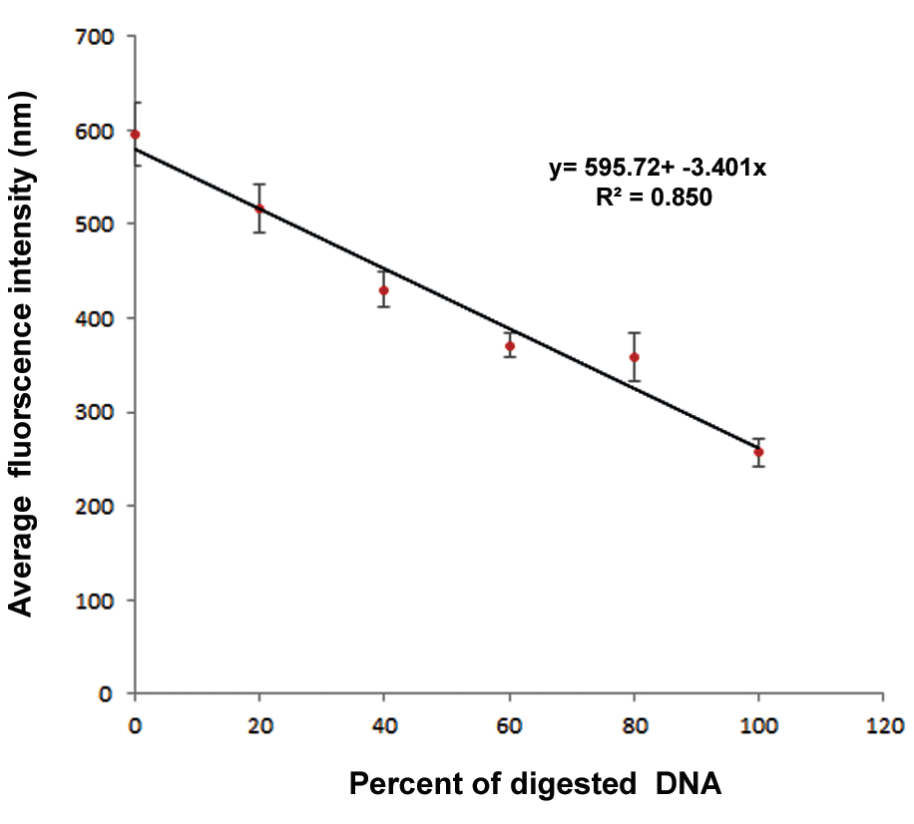
Calibration curve obtained by the amount of fluorescence intensities of control group and DNase- treated group. 3.4 fold change in amount of fluorescence intensity indicates 1% break in DNA. y; Average fluorescence intensity, x; Percent of digestion DNA and R^2^; Regression coefficient.

To determine the percent of induced SSB and DSB in DNA, a calibration curve was drawn. To plot the curve, the average amount of fluorescence intensity was calculated from control and DNase treated groups. The average fluorescence intensity in control group in comparison with treated groups was significantly different (P<0.05). Decrease in fluorescence intensity indicated variation in DNA breaks. Gradient of the linear phase of the curve showed 1% break in DNA. The difference of intensity per break in the DNA strand was equivalents to 341.5, which means, for every 3.415≈3.4 changes in amount of fluorescence intensity, a 1% break occurs. The plotted calibration curve is shown in figure 2.

**Fig.3:**
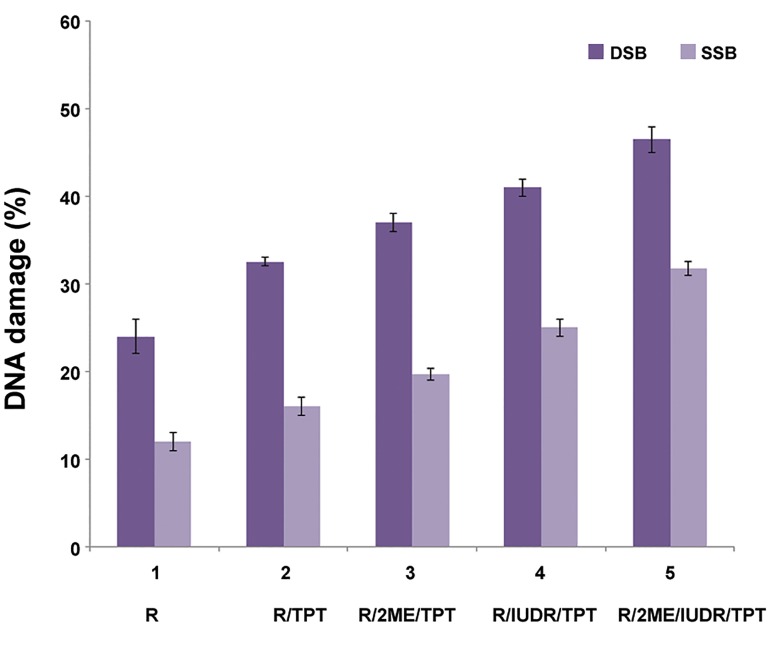
Distribution of average percentage of SSB and DSB in GBM cell groups irradiated (2Gy) and treated with 2ME2/IUDR/TPT. SSB; Single strand break, DSB; Double strand break, GBM; Glioblastoma, 2ME2; 2-Methoxy estradiol, IUDR; Iododeoxyuridine, TPT; Topotecan and R; Radiation.

The difference between average intensities in the control and treated groups was 3.4-fold. Figure 3 display the distribution of the percentage of SSB and DSB in DNA. Combination of R/TPT increased the DSB and SSB in comparison with the group of R (P<0.05). Moreover, successive treatment of cells with R/2ME2/TPT can significantly increase the DSB and SSB when compared with the R group (P<0.05). The comparison of combination of treated cell with R/IUDR/TPT and the R group alone is shown in figure 3. Furthermore, DSB and SSB significantly increased in the presence of TPT with irradiation of X-6 MV after incubation with 2ME2+IUDR when compared with the other four groups R, R/TPT, R/TPT/2ME2 and R/IUDR/TPT (P<0.05). Table 3, shows an increase in DNA damage percentage of 300 μm spheroids in the three groups of R/TPT/2ME2, R/TPT/IUDR and R/TPT /2ME2/IUDR in comparison with the R/TPT group. The effect of combined treatment with R/TPT/2ME2/IUDR was greater than the sum of the effects in two groups of stradiol R/TPT/2ME2 or R/TPT/IUDR.

**Fig.4:**
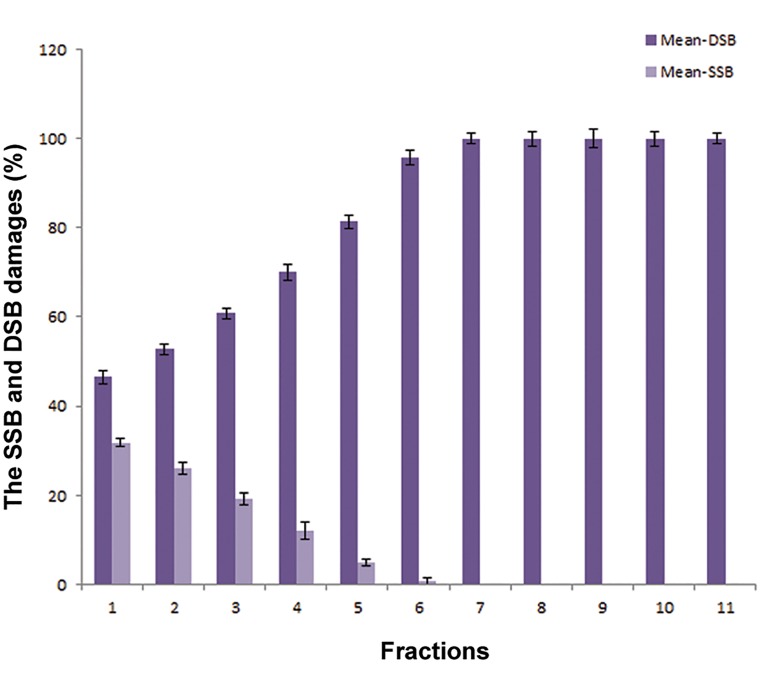
Distribution of average percentage of SSB and DSB of GBM cells in the irradiated [(22Gy (2Gy/fraction)] and the treated groups with 2ME2/IUDR/TPT (twice). SSB; Single strand break, DSB; Double strand break, GBM; Glioblastoma, 2ME2; 2-Methoxy estradiol, IUDR; Iododeoxyuridine and TPT; Topotecan.

In figure 4, successive double treatment of cells with 2ME2/IUDR/TPT and fractional radiation with total dose of 14 Gy (2 Gy/fraction) by 6 MV X-ray can significantly increase the DNA damage in comparison with single treatment and irradiated groups (P<0.05). In the other words, by repeating irradiation from 1 to 7 fractions and repeating drug treatment twice, SSB was reduced while we observed an increase in DSB in DNA. In other words, the SSB converted to the DSB in fractional radiotherapy with X-ray.

**Table 3:** An increase in DNA damage percentage of 300 µm spheroids in three groups of 2ME2/R/TPT, IUDR/R/TPT and 2ME2/IUDR/R/TPT in comparison with the R/TPT group


Increase in DNA damage percentage in group of 2ME2/R/TPT in comparison with R/TPT	Increase in DNA damage percentage in group of IUDR/R/TPT in comparison with R/TPT	Increase in DNA damage percentage in group of 2ME2/IUDR/R/TPT in comparison with R/TPT

8.20%	17.50%	29.83%


2ME2; 2-Methoxy estradiol, IUDR; Iododeoxyuridine, TPT; Topotecan and R; Radiation.

**Fig.5:**
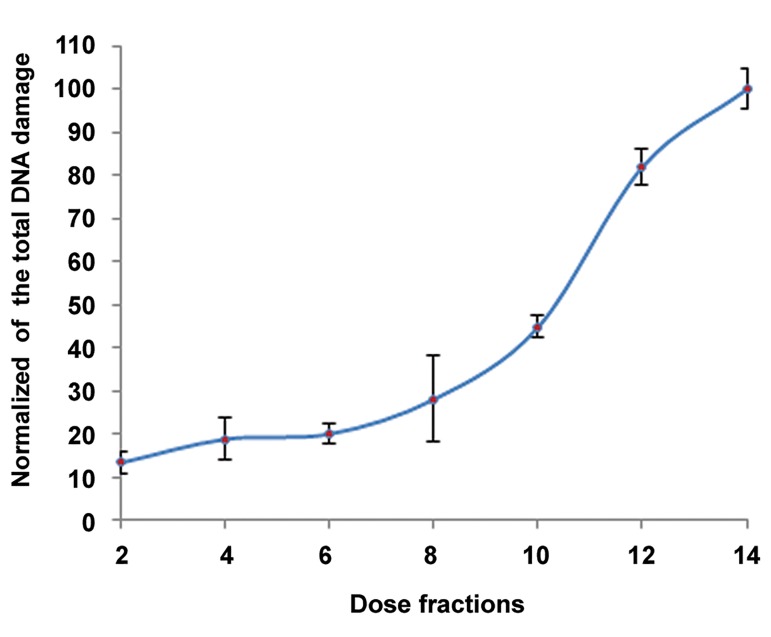
Dose-response effects of X-ray on DNA damage assessed by the Picogreen in the irradiated [14Gy (2Gy/fraction)] and treated groups with 2ME2/IUDR/TPT (twice). An average of nine counts was used to define each point. Mean ± SEM of three experiments is shown on the curve. 2ME2; 2-Methoxy estradiol, IUDR; Iododeoxyuridine, TPT; Topotecan and SEM; Standard error of mean.

The total DNA damage dose-effect relationship was found to be linear in the radiation treatment group with fraction range of 1 to 7 (2 Gy/fraction). Moreover intensity of the damage in the seventh fraction of irradiation reached the ultimate limit (%100). DNA damage was significantly different in all treated and irradiated groups before the sixth fraction when compared with treated and irradiated group at the sixth fraction (P<0.05). We used the biologically effective dose (BED) formulae to determine the α/β ratio of 2 Gy in alternative fractions (Fig.5). The α/β ratio calculated by equation BED50% was 7.36 Gy.

## Discussion

IUDR as a radiosensitizer is more absorbed by cancer cells with high level of proliferation opposed to healthy tissues with low rate of cell division (12). As the absorption of IUDR increases, the level of single and double breaks increases. Therefore, the best choice for assessing these sensitizers is cancer cells with high cell division rate which are surrounded by healthy indivisible cells. Thus glioma are considered to be the best choice since these tumor cells are surrounded by healthy nerve cells with no ability to divide (13). 2ME2 is a potential therapeutic agent for multiple types of tumor including GBM (24-26). It leads to the inhibition of the cell cycle checkpoints in G0/G1 phases (24). Experiments have shown that damages caused by ionizing irradiation after incubation with 2ME2 and IUDR in spheroids treated with 2ME2 is far more than employing IUDR alone (27, 28). Moreover, 2ME2 causes interruption in cell cycle in G2/M phases by affecting microtubules and prevents cancer proliferation. Thus, 2ME2 caused increase in DNA damage as a radiosensitizer (29). In prior studies, 2ME2 was shown to block the growth of colon carcinoma cells and to induce apoptosis (30). In another study, 2ME2, as a potential cytostatic drug, increased autophagic cell death in glioma cells (29). TPT is a radiosensitizer and more recently has been studied *in vitro* and *in vivo* as a chemotherapeutic drug (7, 31, 32).

The Topo-I inhibitor in TPT is a nuclear enzyme that plays a crucial role in DNA replication and in transcription (33). A recent study has shown that nano-liposomal topotecan increased DNA strand breaks and the activity of caspase-3 (marker of programmed cell death) by convection-enhanced in brain tumors (34). In another study genstein and TPT induced cellular death in LNCap cells (prostate cancer) by inhibiting topo- I, II enzyme (35).

In this study TPT was widely used along with other radiosensitizer like 2ME2 and IUDR. Our previous study show that combination of ionizing radiation and TPT could cause synergistic cell killing in various human cancer cells even from highly radioresistant tumors (31). The goal of radiotherapy treatment is to irradiate the tumoral lesion while minimizing the undesirable effects on the neighboring tissues. One of the techniques based on this notion is the technique of fractional radiotherapy. In this method, the carcinogenic cell cannot revive as quickly as the normal cells (36). The processes have been described by the four Rs of radiobiology: as repair, re-distribution in the cell cycle, re-population and re-oxygenation (37). Repair and re-population spare normal tissues in the fractional radiotherapy whereas re-oxygenation and re-assotment increase damages to tumor (38). Therefore this method could be effective in tumor treatment. Combs et al. (39) reported that fractionated radiation with X-rays and carbon ions enhanced the lethal effects of radiation in GBM.

Our results revealed that combination of R/2ME2/IUDR/TPT could significantly increase DSB and SSB in DNA molecules compared with the four groups R, R/TPT, R/TPT/2ME2 or R/IUDR/TPT.

IUDR at the time of cell cycle’s synthesis stage replaces thymidine in DNA threads and increases damage and cell destruction (13). 2ME2 prevented cells from entering the G0 phase, increased IUDR uptake and also caused more sensitivity to radiation treatment (24, 28). Finally by employing TPT, with the capability to inhibit topo-I enzyme, higher level of DNA damage was induced by radiation with X-ray after incubation with 2ME2 and IUDR (33). With fractionated irradiation from 1 to 7 fractions and double drug treatment, SSB was converted to DSB. Zhang et al. (40) reported that TPT combined with chronoradiotherapy could enhance the radiosensitivity of human nasopharyngeal carcinoma. Khoei et al. (28) reported that increase of incubation time from 1 to 2- VDT in 2ME2 treated spheroids produced DNA damage in the cells. Fractionated radiation therapy is an important way of achieving more severe cellular damage due to increasing DSB and complex damage in DNA. Dose-response effects of R/2ME2/IUDR/TPT on GBM damage were assessed by the Picogreen assay in 7 fractions (2 Gy/fraction). The α/β ratio calculated by BED formulae was 7.36 Gy. Previous study have reported the median α/β ratio of 5.43 Gy from GBM cells after 2 Gy x-ray exposure (41). Increasing the ratio of α/β from 5.43 in R group to 7.36 Gy in R/2ME/IUDR/TPT groups from GBM cell line U87 seemed to be reasonable, because with increasing DSB, repair of this damage hardly occurred (42, 43). In another study, the median α/β ratio for temozolomide/radiation (TMZ/R) was 9.32 Gy in the GBM-U87 cell line (44). Furthermore, the DNA damage was greater in the presence of R/ 2ME2/TPT compared with R/TPT alone. This could be due to cease of cell cycles in G2/M phases and thus prevention of tumor growth. That is why in this study, in addition to TPT we used IUDR and 2ME2 as radiosensitizer agents so that their combined use would have an accumulative effect especially in fractionated radiotherapy. With this accumulative effect, the absorption dose to create damage will be increased significantly and treatment of GBM could have more success.

## Conclusion

These results suggest that R/TPT-treated cells increase sensitivity of 2ME2 and IUDR especially when they are used together and may improve the therapeutic index for radiation. Our purpose for further studies is to make use of TPT with pifithrin or inhibitors of p53 as a co-treatment after irradiation with X-6MV to enhance tumor radiosensitization to TPT and then evaluate the combined effects of these agents on the cells.
